# Accuracy of Noninvasive Hemoglobin and Venous Blood Gas Hemoglobin Measurements in Comparison With the Laboratory Method in an Intensive Care Unit

**DOI:** 10.7759/cureus.102698

**Published:** 2026-01-31

**Authors:** Marwa M Elmaghrabi, Afrah Alatifi, Najla Alsoofi, Nancy Ahmed, Sofia Rauf, Mohamed T Yassin, Osama Sobh

**Affiliations:** 1 Botany and Microbiology Department, College of Science, King Saud University, Riyadh, SAU; 2 Microbiology Department, Center of Excellence for Research in Regenerative Medicine and Applications (CERRMA) Faculty of Medicine, Alexandria University, Alexandria, EGY; 3 Critical Care Department, King Saud Hospital, Al Qassim, SAU; 4 Internal Medicine/Rheumatology Department, King Saud Hospital, Al Qassim, SAU; 5 Critical Care Department, Intensive Care Unit, King Saud Hospital, Al Qassim, SAU

**Keywords:** blood loss, blood transfusion, hematology measurements, non-invasive hemoglobin, venous blood gas

## Abstract

Background

Rapid and accurate hemoglobin (Hb) measurement is crucial in Intensive Care Unit (ICU) patients for timely management of anemia and transfusion decisions. Noninvasive hemoglobin monitoring (SpHb) offers continuous assessment, while venous blood gas hemoglobin (VBGHb) provides point-of-care measurement. This study aimed to evaluate the absolute and trend accuracy of SpHb and VBGHb compared to standard laboratory hemoglobin (LabHb) in ICU patients.

Methods

Twenty patients in the ICU were enrolled. Hemoglobin was measured using SpHb, VBGHb, and LabHb. Absolute accuracy was assessed using Bland-Altman analysis, and correlation between methods was evaluated with Pearson correlation. Trend accuracy was assessed with modified Bland-Altman plots.

Results

A total of 79 samples were collected. SpHb demonstrated a mean bias of −0.0557 gm/dL (95% limits of agreement: −0.5614 to 0.4499) relative to LabHb, while VBGHb showed a larger bias of −0.4734 gm/dL (−2.8312 to 1.8844). SpHb had a strong correlation with LabHb (r = 0.978, p < 0.001), whereas VBGHb showed a moderate correlation (r = 0.506, p < 0.001). Trend analysis revealed a mean trend bias of −0.0102 gm/dL for SpHb (limits −0.6317 to 0.6113), compared with 0.0136 gm/dL for VBGHb (−3.5733 to 3.5461).

Conclusions

We concluded that SpHb demonstrated acceptable absolute and trend accuracy, as well as excellent correlation with LabHb, providing an easy, feasible, and accurate solution for Hb measurement in the ICU. Future studies should assess its impact on transfusion practices and patient outcomes.

## Introduction

One of the major influences of technology on patient care has been the shift in paradigm from highly invasive diagnostic and therapeutic procedures to non-invasive ones. This has reduced the time of diagnosis, critical decision-making, and treatment, and improved patient satisfaction. Hemoglobin (Hb) measurement is one of the most frequently performed laboratory tests after injury [1]. Noninvasive hemoglobin (SpHb) monitoring is a more recent introduction to the growing list of point-of-care testing capabilities that allow for the ability to monitor hemoglobin concentration in a continuous, accurate, and non-invasive fashion [2]

Prompt and ongoing evaluation of total hemoglobin levels is essential for accurately assessing blood loss, the need for transfusion, and the hidden bleeding [3,4]. Traditional methods for confirming hypovolemia, such as hemoglobin and hematocrit measurements, are not always available at the point of care, and hemodynamic monitoring may not effectively detect significant blood loss. Delays in laboratory results may negatively impact patient outcomes or diagnostic studies [5-7].

Accurately measuring blood hemoglobin is crucial for managing both chronic and acute blood loss. However, invasive methods can have drawbacks, including the consumption of valuable time, the risk of phlebotomy-induced anemia, pain, potential for infection, and increased demand for human resources and equipment [8,9].

Masimo Corporation (Irvine, CA, USA) has developed an innovative pulse CO-oximeter that measures hemoglobin concentration using a non-invasive, multi-wavelength sensor for both spot checking and continuous monitoring. The technology depends on emitting multiple wavelengths of light and then calculating the hemoglobin concentration based on light absorption in the blood. The device uses a finger probe similar to a regular pulse oximeter sensor and reads the hemoglobin noninvasively [10]

This study aims to evaluate the absolute and trend accuracy of SpHb and venous blood gas hemoglobin (VBGHb) measurements compared to standard laboratory hemoglobin (LabHb) in the ICU.

## Materials and methods

Study design and population

Twenty adult patients in the ICU were enrolled, and their demographic data, including age, sex, and primary diagnosis, were recorded. It was done on all adult patients who were admitted to the ICU with bleeding and needed blood transfusion from October 2022 to December 2022 (septic shock patients with disseminated intravascular coagulation (DIC) were included).

This convenience sample observational study received Regional Research Ethics Committee approval (No. 607/44/10046) at King Saud Hospital, Unaizah.

Inclusion and exclusion criteria

The inclusion criteria for this study consisted of adult patients aged 18 years and older who were admitted to the intensive care unit (ICU). This group included patients experiencing acute bleeding requiring blood transfusions, as well as those diagnosed with septic shock and DIC. 

The exclusion criteria included pediatric patients under 18 years of age, individuals with incomplete hemoglobin data, and patients whose poor signal quality negatively impacted non-invasive hemoglobin monitoring.

Hemoglobin measurement

Non-invasive hemoglobin (SpHb) measurements were obtained using a Masimo pulse CO-oximeter (Radical-7®, Masimo Corporation, Irvine, CA, USA) equipped with multi-wavelength disposable finger sensors, which were placed on the ring finger of patients for continuous SpHB monitoring versus the results of blood samples where hemoglobin (Hb) was measured by standard lab Hb using a clinical laboratory hematology analyzer (LabHb; ADIVA® 2120i, Siemens Healthineers, Erlangen, Germany). The SpHb sensor was placed on the ring finger of each patient to allow continuous noninvasive hemoglobin monitoring. These SpHb measurements were compared with Hb values obtained from venous blood samples (VBGHb). Venous blood gas hemoglobin (VBGHb) was measured using a blood gas analyzer.

Four venous blood samples were collected from each patient simultaneously during routine blood sampling. At the time of blood withdrawal, SpHb measurements were taken through continuous, noninvasive monitoring using a pulse CO-oximeter. The measurements were recorded exactly when the venous blood samples were collected. These samples were subsequently analyzed for hemoglobin concentration using both a clinical laboratory hematology analyzer (LabHb) and a venous blood gas analyzer (VBGHb). This method allowed for a comparison of hemoglobin measurements obtained at the same time using different techniques.

Each measurement modality was analyzed using its respective analyzer, and samples were not run interchangeably across different devices. When applicable, duplicate measurements were performed only within the same analyzer to ensure analytical reliability and were not used to assess agreement between different measurement methods.

Data analysis

Absolute accuracy was assessed using Bland-Altman analysis. Correlation between methods was calculated with Pearson correlation. Trend accuracy was assessed using modified Bland-Altman analysis.

Statistical Methods

All analyses were performed using SPSS (Statistical Package for the Social Sciences) software (v.26.0; IBM Corp., Armonk, USA). Data were presented as mean ± standard deviation (SD) for numerical variables and frequency (%) for non-numerical variables. The Pearson correlation coefficient (r) was used to assess correlations among SpHb, VBGHb, and LabHb measurements, and a linear regression curve was used to assess the relationship between SpHb and VBGHb. 

Bland-Altman analysis was performed to estimate the mean bias and the degree of agreement between the two measures. Modified Bland-Altman analysis of the difference between changes in SpHb, VBGHb, and LabHb as the measurement standard, to obtain bias and 95% limits of agreement between trends in SpHb, VBGHb, and LabHb.

Trend analysis was defined as the change in hemoglobin values between consecutive blood samples collected sequentially over time from the same patient. Each blood sample represented a distinct time point and was analyzed once using each measurement method. Changes in Hb between paired sequential samples were compared between SpHb, VBGHb, and laboratory hemoglobin measurements to assess the ability of each method to track directional changes over time, rather than analytical repeatability. We included in the analysis only samples for which all measures were available for trend calculations.

## Results

Patient demographics and clinical characteristics

Twenty ICU patients were enrolled in the study. Thirteen (65%) were male, and seven (35%) were female. Their ages ranged from 22 to 78 years, with a mean of 49.25 ± 15.92 years. Primary diagnoses included road traffic accidents with hemorrhagic shock (n = 9, 45%), gastrointestinal bleeding (n = 5, 25%), and septic shock with DIC (n = 6, 30%) (Table 1).

**Table 1:** Demographic and clinical characteristics of the study population. Age is presented as mean ± SD with range. Gender and primary diagnoses are clearly organized. SpHb: noninvasive hemoglobin; LabHb: laboratory hemoglobin; DIC: disseminated intravascular coagulation

Characteristic	Value
Age (years)	49.25 ± 15.92 (range: 22–78)
Male	13 (65%)
Female	7 (35%)
Road traffic accident with hemorrhagic shock	9 (45%)
Gastrointestinal bleeding	5 (25%)
Septic shock with DIC	6 (30%)

Primary diagnosis

The primary diagnosis included road traffic accidents with hemorrhagic shock in nine patients (45%), gastrointestinal bleeding in five patients (25%), and septic shock with disseminated intravascular coagulation (DIC) in six patients (30%).

Absolute accuracy of SpHb and VBGHb

A total of 79 hemoglobin samples were collected. The mean hemoglobin values measured by SpHb, VBGHb, and LabHb are shown in Table 2.

**Table 2:** Hemoglobin measurements in ICU patients The table summarizes hemoglobin measurements collected from 20 critically ill ICU patients (79 total samples). Data are presented as mean ± standard deviation, with ranges shown in parentheses. SpHb: noninvasive hemoglobin measured by pulse co-oximetry; VBGHb: hemoglobin measured by venous blood gas analysis; LabHb: hemoglobin measured by standard laboratory hematology analysis (reference standard).

Measurements	Results
SpHb	(2.6-10.1) 7.94 ± 1.23 gm/dL
VBGHb	(3.8-10.6) 8.35 ± 1.22 gm/dL
LabHb	(2.4- 10) 7.88 ± 1.19 gm/dL

**Figure 1:**
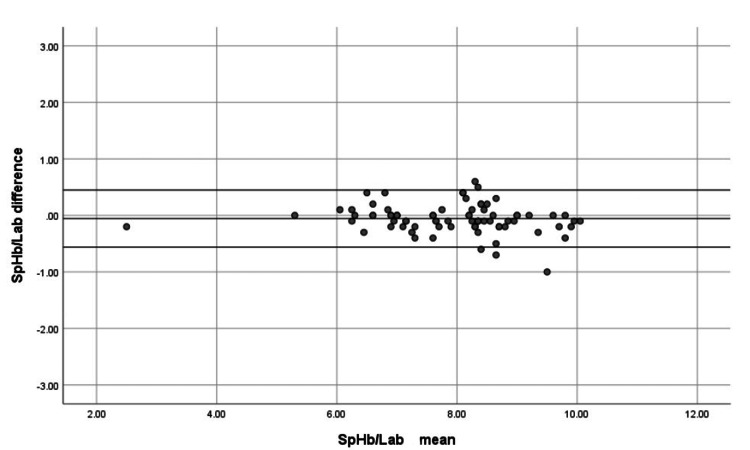
Bland-Altman plot comparing noninvasive hemoglobin (SpHb) and laboratory hemoglobin (Lab) measurements Mean bias −0.0557 gm/dL, with 95% limits of agreement −0.5614 to 0.4499 gm/dL, indicating excellent absolute accuracy.

**Figure 2:**
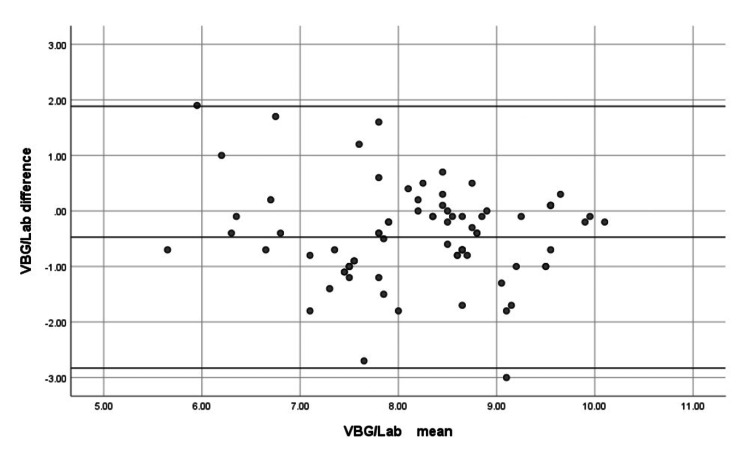
Bland-Altman analysis comparing VBGHb with LabHb demonstrates greater bias and wider limits of agreement. LabHb is used as the reference standard, which allows inference of lower accuracy and higher variability of VBGHb relative to the reference method. VBG/VBGHb: venous blood gas hemoglobin; Lab/LabHb: laboratory hemoglobin

Bland-Altman analysis showed that SpHb had a mean bias of −0.0557 gm/dL with 95% limits of agreement from −0.5614 to 0.4499 gm/dL (Figure 1), indicating excellent absolute accuracy. VBGHb displayed a larger mean bias of −0.4734 gm/dL with wider 95% limits of agreement (−2.8312 to 1.8844 gm/dL) (Figure 2), reflecting lower accuracy.

Bland-Altman analysis showed that VBGHb had greater bias and wider limits of agreement when compared with LabHb. Since LabHb was considered the reference standard, these results indicate lower accuracy and higher variability of VBGHb relative to the reference method.

**Figure 3:**
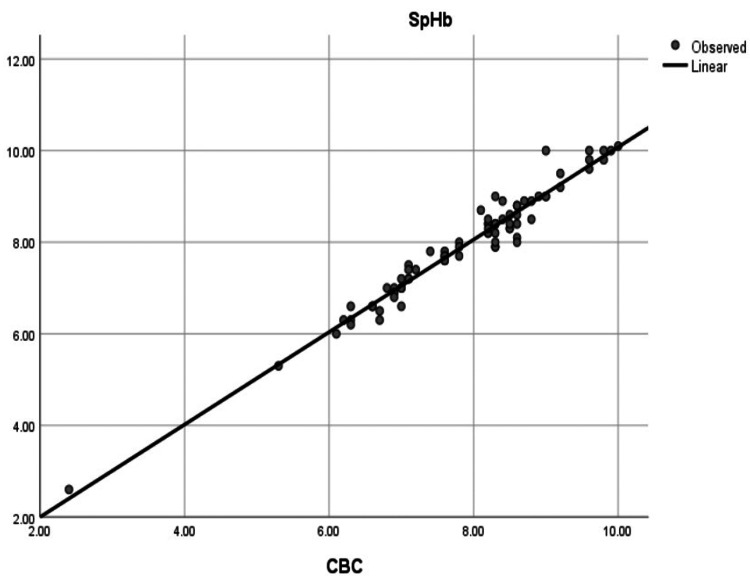
Correlation between SpHb and LabHb. Scatter plot showing strong correlation (r = 0.978, p < 0.001) between SpHb and LabHb. SpHb: noninvasive hemoglobin; LabHb: laboratory hemoglobin; CBC: complete blood count

**Figure 4:**
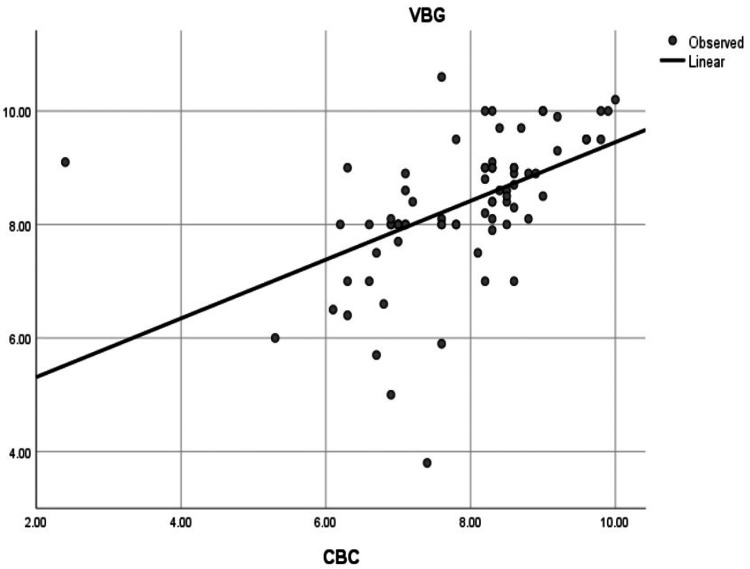
Correlation between VBGHb and LabHb. Scatter plot showing moderate correlation (r = 0.506, p < 0.001) between VBGHb and LabHb. VBG/VBGHb: venous blood gas hemoglobin; LabHB: laboratory hemoglobin; CBC: complete blood count

Pearson correlation demonstrated a very strong correlation between SpHb and LabHb (r = 0.978, p < 0.001) (Figure 3) and a moderate correlation between VBGHb and LabHb (r = 0.506, p < 0.001) (Figure 4).

We found a highly significant correlation between SpHb and LabHb, as indicated by the Pearson Correlation coefficient (r = 0.978, p < 0.001), as shown in Figure 3. Additionally, a highly significant correlation was found between VBGHb and LabHb (r = 0.506, p < 0.001), as shown in Figure 4.

Trend accuracy of SpHb and VBGHb

**Figure 5:**
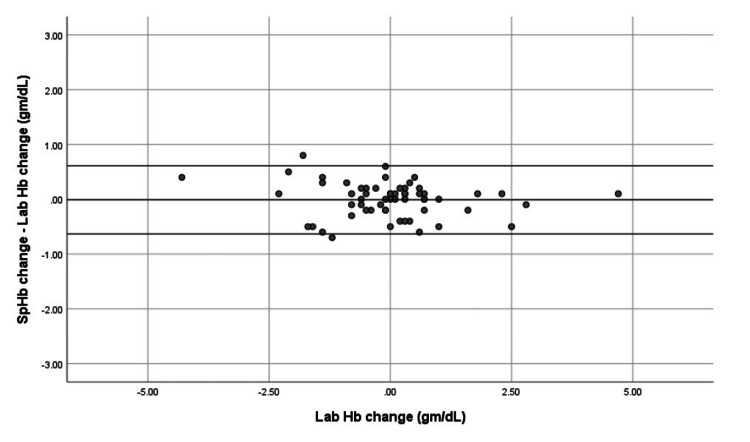
Trend analysis of SpHb versus LabHb using a modified Bland-Altman plot. SpHb values were obtained as spot measurements at the time each venous sample was drawn. LabHb values represent the mean of duplicate measurements from the same sample to reduce analytical variability. Mean trend bias was −0.0102 g/dL, with limits of agreement −0.6317 to 0.6113 g/dL, demonstrating that SpHb reliably tracks hemoglobin changes over time. SpHb: noninvasive hemoglobin; LabHb: laboratory hemoglobin

**Figure 6:**
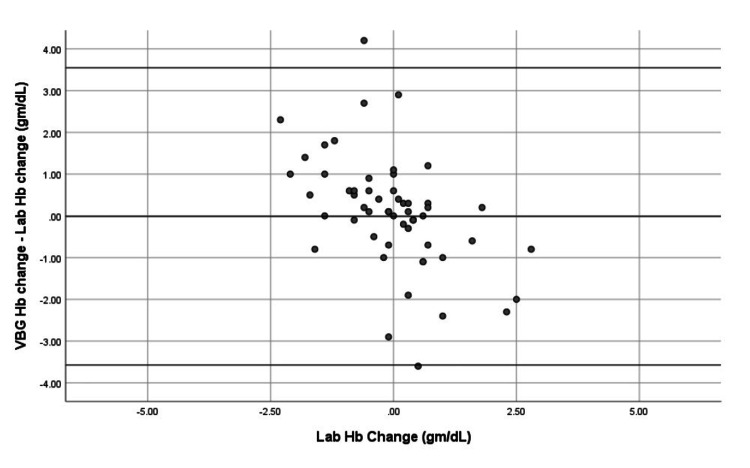
Trend analysis of VBGHb versus LabHb by Modified Bland-Altman plot of sequential measurements. Mean trend bias was 0.0136 gm/dL with limits of −3.5733 to 3.5461 gm/dL, indicating greater variability than SpHb. VBGHb: venous blood gas hemoglobin; LabHb: laboratory hemoglobin; SpHb: noninvasive hemoglobin

Trend accuracy of SpHb and VBGHb was assessed using modified Bland-Altman analysis. SpHb values were recorded as spot measurements at the time each venous sample was drawn, while LabHb values represent the average of duplicate measurements from the same sample to minimize analytical variability. SpHb demonstrated a mean trend bias of −0.0102 g/dL (limits −0.6317 to 0.6113 g/dL), indicating reliable tracking of hemoglobin changes over time (Figure 5). VBGHb showed a mean trend bias of 0.0136 g/dL (limits −3.5733 to 3.5461 g/dL) (Figure 6), indicating greater variability.

These results suggest that SpHb can be used for both accurate point-of-care measurement and dynamic hemoglobin monitoring in ICU patients, whereas VBGHb shows more inconsistency.

Summary of key findings

Absolute accuracy analysis showed that SpHb had a mean bias of −0.056 g/dL with limits of agreement ranging from −0.5614 to 0.4499 g/dL. In contrast, VBGHb demonstrated a mean bias of −0.4734 g/dL with wider limits of agreement from −2.8312 to 1.8844 g/dL.

Correlation analysis demonstrated a strong correlation between SpHb and laboratory hemoglobin (LabHb) measurements (r = 0.978, p < 0.001), while the correlation between VBGHb and LabHb was moderate (r = 0.506, p < 0.001).

Trend accuracy analysis revealed that SpHb exhibited a mean trend bias of −0.0102 g/dL with limits of agreement from −0.6317 to 0.6113 g/dL, whereas VBGHb showed a mean trend bias of 0.0136 g/dL with wider limits of agreement ranging from −3.5733 to 3.5461 g/dL.

## Discussion

In the present study, we found a very low bias between non-invasive hemoglobin measurements (SpHb) and laboratory hemoglobin (LabHb), with a mean bias of −0.0557 gm/dL and 95% limits of agreement ranging from −0.5614 to 0.4499 gm/dL. This indicates excellent absolute accuracy of SpHb in ICU patients.

Previous studies in critically ill patients have reported higher bias for SpHb measurements. For example, a study on ICU patients (Population (P): critically ill patients; Intervention (I): SpHb; Comparator (C): LabHb; Outcome (O): higher bias than current study) reported wider limits of agreement [11]. Similarly, studies conducted on trauma patients (P: trauma patients in emergency department (ED); I: SpHb; C: LabHb; O: clinically unacceptable accuracy) found that the accuracy of SpHb was not always clinically acceptable [12,13]. However, a recent study conducted in Egypt on 70 major trauma patients with low hemoglobin levels (P: trauma patients with low Hb; I: SpHb; C: LabHb; O: clinically acceptable bias and limits of agreement) demonstrated better accuracy, consistent with our findings [14].

Compared to Applegate et al. (2020) (P: surgical patients; I: SpHb; C: LabHb; O: bias 0.24 gm/dL, limits −2.05 to 2.53), our study showed smaller bias and narrower limits of agreement [15]. The higher variability in their study may be attributable to the use of arterial samples for analysis. Our results are consistent with Gamal et al., 2017 (P: trauma patients; I: SpHb; C: LabHb; O: mean bias 0.12, limits −0.56 to 0.79), who used venous sampling similar to our study [14].

Regarding correlation, we found a highly significant correlation between SpHb and LabHb (r = 0.978, p < 0.001). This finding aligns with Adel et al. (2018) (P: ICU/surgical patients; I: SpHb; C: LabHb; O: r = 0.938), who reported an excellent correlation [16]. Our results are also supported by two studies conducted in emergency departments: Osborn et al. (2019) (P: ED patients; I: SpHb; C: LabHb; O: r = 0.77) and Al Aseri et al. (2022) (P: ED patients; I: SpHb; C: LabHb; O: r = 0.812), both of which found highly significant correlations between SpHb and LabHb [17,18].

We further evaluated the trend accuracy of SpHb and VBGHb using modified Bland-Altman analysis. SpHb demonstrated a mean trend bias of −0.0102 gm/dL, with limits of agreement from −0.6317 to 0.6113 gm/dL, while VBGHb showed a mean trend bias of 0.0136 gm/dL with much wider limits of −3.5733 to 3.5461 gm/dL. These results are consistent with Applegate et al. (2020) (P: surgical patients; I: SpHb; C: LabHb trend; O: trend bias 0.0055, limits −0.25 to 0.26) for SpHb trend accuracy. The wider variability in blood gas hemoglobin trends reported by Applegate et al. (bias −0.032; limits −0.61 to 0.54) may be attributed to arterial sample usage [15]. Similarly, Gamal et al., 2017 (P: trauma patients; I: SpHb trend; C: LabHb trend; O: mean trend bias −0.05, limits −0.62 to 0.51) reported narrow limits of agreement for SpHb trend changes, consistent with our findings [14].

One recent study concluded that Hb measured by ABG (arterial blood gas) analysis was significantly higher than that measured by the lab method [19]. Overall, our findings suggest that SpHb is not only highly accurate for absolute hemoglobin measurement but also reliable for continuous trend monitoring in ICU patients. In contrast, VBGHb demonstrated greater variability in both absolute and trend measurements, which may limit its utility for transfusion decision-making in critically ill patients.

As noted by Applegate et al., the similar agreement in trend direction between SpHb and laboratory measurements suggests that clinicians can choose the method based on availability or preference, although continuous SpHb monitoring may provide useful ongoing hemoglobin trend information [15].

Limitations

This study has several limitations. First, the sample size was small (20 patients), which may limit the generalizability of the findings. Second, it was conducted at a single ICU setting, and results may differ in other clinical environments. Third, only adult patients were included, and pediatric patients were excluded. Fourth, patients had different shock states, including hemorrhagic and vasodilatory shock, which may introduce variability in SpHb accuracy due to differences in peripheral perfusion. Fifth, the study did not control for potential confounders known to affect pulse oximeter readings, including skin tone, body temperature, body habitus, and tissue thickness. Sixth, patients had a wide age range and diverse comorbidities, which may influence circulatory status and device accuracy. Finally, patients with extreme hemoglobin values were rarely included, limiting the assessment of device performance across the full Hb spectrum.

Despite these limitations, the study population reflects real-world ICU conditions, and the results provide valuable insights into the performance of non-invasive hemoglobin monitoring in critically ill patients.

Future studies are warranted to evaluate the effect of SpHb monitoring on blood transfusion practices, patient outcomes, and cost-effectiveness in critically ill patients.

## Conclusions

In this study, SpHb demonstrated acceptable absolute accuracy and reliable trend performance when compared with LabHb measurements in critically ill patients admitted to the intensive care unit. SpHb showed a strong correlation with LabHb and a lower measurement bias with narrower limits of agreement than VBGHb, suggesting better analytical agreement with the reference laboratory method.

The ability of SpHb to track hemoglobin changes over time supports its use as a continuous, non-invasive monitoring modality in the ICU. This may be particularly valuable in patients requiring frequent hemoglobin assessment, as it can potentially reduce repeated blood sampling and provide timely information to support clinical monitoring.

Nevertheless, SpHb should be used as an adjunct to standard laboratory testing rather than a replacement, especially when precise hemoglobin values are required for transfusion decisions. These findings are based on a limited number of ICU patients with blood loss requiring transfusion and a specific range of hemoglobin values; therefore, caution should be exercised in generalizing these results to all critically ill ICU patients. Larger, multicenter studies are needed to further validate the accuracy of SpHb and its impact on transfusion practices and clinical outcomes.
